# Supplementary material for: Analysis of very-high surface area 3D-printed media in a moving bed biofilm reactor for wastewater treatment
Source: PLoS One. 2020 Aug 27;15(8):e0238386. doi: 10.1371/journal.pone.0238386 (PMC7451639; doi:10.1371/journal.pone.0238386)
Supplement: Attachment — Submitted filename: PONE-D-20-13545.docx [file pone.0238386.s001.docx]

**PONE-D-20-13545**

Analysis of Very-High Surface Area 3D-printed Media in a Moving Bed Biofilm Reactor for Wastewater Treatment

The study is about the wastewater treatment specific to ammonia/TAN using a high surface area 3D fabricate gyroid-shaped bio-carrier. Treatment performance was compared with the market available commercial bio-carrier. Wastewater treatment results in terms of ammonia removal are interesting with 3D fabricated gyroid-shaped bio-carriers compared to conventional bio-carrier. However, the experiments were carried out using synthetic wastewater. The actual performance/potential of bio carriers can be examined with real field wastewater even at the lab scale. The discussion on removal is weak, not focused the treatment efficiency.

**Comments**

- The photograph showed for commercial bio-carrier is actually not such bad as shown here, it can be accepted that 3D-Printed bio carrier is better but portraying such images for commercial bio-carrier is not acceptable.
- Real field wastewater treatment is always advisable to evaluate the potential of designed bio carriers. The process may be examined with real field wastewater if possible.
- What is the life of designed bio carriers and their interaction with microbes?
- Quality of graphs are very poor to read the contents.
- Why the initial concentration of nitrates and TAN was chosen below 5 mg/L as wastewater typically contains more than 20 mg/L.
- Any figure can be produced with biofilm formation on designed and conventional bio carrier.
- Ammonia, ammonium, nitrite, nitrate, pH, chlorine, hardness, and alkalinity were also inspected but values were not mentioned in the study.
- Line 303: it cannot be claimed that SSA is the main parameter for maximizing the performance of MBBRs as many other factors influence the whole treatment process.
- Ammonia removal close to 100% might be due to the adaption of microbes at low concentration of ammonia being fed in the bioreactor- the case might be opposite with real field wastewater or when the concentration of ammonia is more than 5 mg/L.
- Any relation found for nitrate production and TAN removal, as nitrate production is almost the same whereas trend for TAN removal is quite opposite.
