# Supplementary material for: Analysis of very-high surface area 3D-printed media in a moving bed biofilm reactor for wastewater treatment
Source: PLoS One. 2020 Aug 27;15(8):e0238386. doi: 10.1371/journal.pone.0238386 (PMC7451639; doi:10.1371/journal.pone.0238386)
Supplement: Attachment — Submitted filename: Response to Reviewers.docx [file pone.0238386.s003.docx]

**Response to Reviewers**

We present the response to reviewers in detail in this communication. We thank the reviewers again for their helpful and constructive comments that continue to improve this manuscript. We have addressed all comments in this letter and/or in the manuscript.

**Reviewer #2**:


*Authors have addressed most of comments. However, figures are presented in different sub-figures (in several pages); it would be more suitable if each figure (with all sub-figures) is included in one page.*

Thank you for your comments and observations on the figures. We have re-combined all the sub-figures as panels in each overall figure, formatted to fit on one page.

• *Table 1 contains several symbols "*" and "**" but the meaning of them is missing.*

Thank you for catching that error resulting from formatting the table for submission. We have now included the table footnotes that are indicated by those symbols (Table 1, p. 7, markup manuscript).

*Finally, when you speak about the surface area (0.055 m2) and the numbers of carriers (7) it is necessary to specify the references used (it is not sufficient to say "preliminary investigation").*

Thank you for your comment. The theoretically required surface area was initially calculated using performance metrics estimated from literature, and then validated through trial-and-error experimentation in preliminary work in the laboratory, the data for which are not reportable. However, the calculations that support the initial planning are reported in a published document. We have included this reference in the text, and indicated more clearly the preliminary aspects of the trials, in the following location (lines 278-280, Manuscript Markup):

**In all experiments, sizing was determined based on calculations of minimum required biocarrier surface area for complete nitrification, as reported in [****36****, p.95] and validated through trial and error in preliminary trials (data not reported here).**

Where the reference that is newly included as a citation (line 629-630, Manuscript Markup):

**Proaño Peña GF. 3D-printed Custom Substratum for Fast Functional Responses from Microbial Colonization. Auburn, Alabama, USA: Auburn University; 2018.**

Also, in the following location (lines 297-300, Manuscript Markup):

**The total surface area per reactor of 0.055 m^2^ was chosen based on the minimum amount of surface area for gyroids needed for a measurable rate of nitrification throughout the time of the trial, as determined through trial and error preliminary investigations and confirmed through repetition in other experiments (for example, Experiment 1, Table 2).**

Also, in the following location (line 310-313, Manuscript Markup):

**The constant count value of 7 units of biocarriers in each reactor was chosen based on the minimum number of Kaldnes K1 biocarriers needed for a detectable amount of total ammonia conversion during the 8-hour trials, as determined through observations in trial and error preliminary investigations (data not included here).**
